# LIM Mineralization Protein-1 Inhibits the Malignant Phenotypes of Human Osteosarcoma Cells

**DOI:** 10.3390/ijms15047037

**Published:** 2014-04-23

**Authors:** Huiwen Liu, Lu Huang, Zhongzu Zhang, Zhanming Zhang, Zhiming Yu, Xiang Chen, Zhuo Chen, Yongping Zen, Dong Yang, Zhimin Han, Yong Shu, Min Dai, Kai Cao

**Affiliations:** 1 Department of Orthopedics, the First Affiliated Hospital of Nanchang University, Nanchang 330006, China; 2 Department of Orthopedics, the First People’s Hospital of Jingdezhen, Jingdezhen 333000, China; 3 Department of Children Health and Care, Jiangxi Maternal and Child Health Hospital, Nanchang 330006, China; 4 Department of Oncology, the First Affiliated Hospital of Nanchang University, Nanchang 330006, China

**Keywords:** *LMP-1*, osteosarcoma, tumor suppressor gene, *Runx2*, *BMP-2*

## Abstract

Osteosarcoma (OS), also known as osteogenic sarcoma, is the most common primary malignancy of bone tumor in children and adolescents. However, its underlying molecular pathogenesis is still only vaguely understood. Recently, LIM mineralization protein-1 (*LMP-1*) was reported to be an essential positive regulator of osteoblast differentiation. In the present study, we found that the expression of *LMP-1* is downregulated in OS tissues compared with adjacent normal tissues. Moreover, we restored the expression of *LMP-1* through a recombinant adenovirus. Overexpression of *LMP-1* inhibited cell proliferation and invasion, arrested cell cycle progression, and induced apoptosis *in vitro*. Finally, ectopic *LMP-1* expression suppressed the expression of *Runx2* and *BMP-2* in OS cells. These data demonstrate that *LMP-1* is an essential tumor suppressor in the OS pathological process, which will provide a new opportunity for discovering and identifying novel effective treatment strategies.

## 1. Introduction

Osteosarcoma (OS) is a primary malignant tumor of the bone, which is characterized by its malignant potential of rapid distal metastasis [1]. Although OS can occur in any parts of bone, it is frequently found in the metaphysis of long bones, especially in the distal femur and proximal tibia during the period of rapid growth [2]. In the past 20 years, with the development of neo-adjuvant therapy, the long-term survival rate of patients with OS has been remarkably improved [3]. However, there are still about 20% of patients with distal metastasis having an extremely poor prognosis, with a long-time survival rate less than 10% [4]. The etiology of this neoplasm is complex, and both genetic and environmental factors contribute to the complicated scenario, which results in a deficiency of specific targets and efficient chemotherapy regimens. Thus, a comprehensive understanding of the biology of this malignancy is definitely necessary for the development of novel therapies.

More and more evidence suggests that OS may be a kind of differentiation disease [5]. Disruption of osteogenic differentiation may lead to the development of OS [6,7], for example, Luo *et al.* [7] found that osteogenic regulators, *Runx2*, *Osterix*, as well as the later marker osteopontin (*OPN*) were all suppressed in the majority of OS cell lines. Moreover, exogenous *Runx2* expression could inhibit the tumor growth of OS cells [7], which suggested that OS cells may be obstructed in undergoing terminal differentiation by impairments in the osteogenic pathway.

LIM mineralization protein-1 (*LMP-1*) is a kind of intracellular non-secreted protein [8]. Recently, numerous studies have demonstrated that *LMP-1* is a key regulator of osteoblast differentiation [8–11]. Overexpression of *LMP-1* induced the mRNA expression of *RunX2* and elevated the alkaline phosphatase (*ALP*) activity of osteoprogenitor cells [12]. Additionally, *LMP-1* retroviral gene therapy was reported to be a better potential candidate for influencing osteoblast differentiation and fracture repair than bone morphogenetic protein-4 (*BMP-4*), which is the most important gene in regulating osteoblast differentiation until now [13]. However, the role of *LMP-1* in OS carcinogenesis is less known. Given the powerful osteogenic functions, we therefore hypothesized that *LMP-1* may play an important role in OS tumorigenicity.

In this paper, we have undertaken studies to explore the expression and involvement of *LMP-1* in OS carcinogenesis. Firstly, we explored the expression of *LMP-1* in 43 OS specimens, and found that the expression of *LMP-1* was significantly suppressed in OS tissues compared with adjacent normal tissues. Subsequently, through infection with recombinant adenovirus AdLMP-1 into the OS cell lines, U2OS and SaOS-2, we completed a series of cellular function experiments to investigate the role of *LMP-1* in OS cells and to identify the mechanisms involved.

## 2. Results

### 2.1. LMP-1 Expression Is Dysregulated in OS Tissues

**Figure 1.**
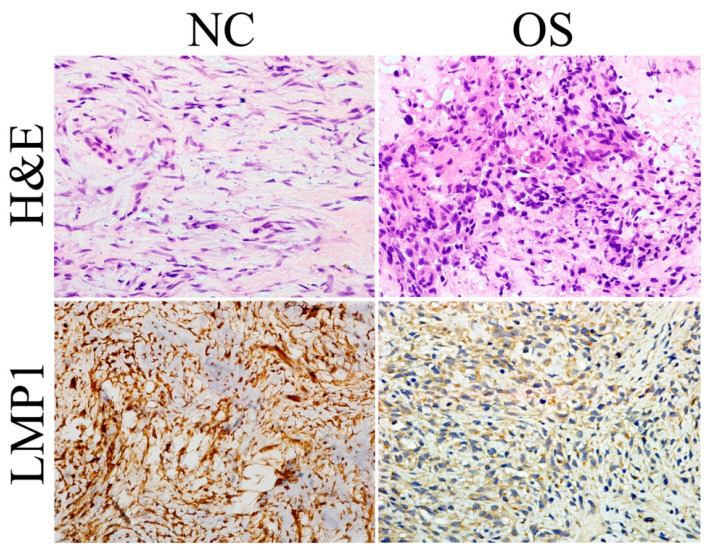
Dysregulated *LMP-1* expression in osteosarcoma (OS) tissues. Representative immunohistochemical images showing LMP-1 expression in primary OS and adjacent normal tissues. Positive cells were scattered throughout the matched normal tissues (**left panel**); 31 out of 43 total OS specimens showed suppressed levels of the LMP-1 protein (scores 0 and 1+, **right panel**). HE, hematoxylin and eosin (×100).

In an attempt to explore the expression and significance of *LMP-1* in OS carcinogenesis, we explored the expression of *LMP-1* in 43 pairs of OS tissues compared with the matched normal tissues. These tissues have been confirmed histologically by hematoxylin eosin (HE) staining (Figure 1). Our results showed that the expression of LMP-1 is mainly distributed in the cytoplasm of cells. Positive cells were scattered throughout the matched normal tissues (Figure 1). However, 31 out of 43 total OS specimens showed suppressed expression of the LMP-1 protein (scores 0 and 1+) (Figure 1). The extent of LPM-1 positive tumor cells amongst diffusely positive tumors was graded as follows: 12 tumors 0; 19 tumors 1+; 8 tumors 2+; 4 tumor 3+. These results suggested that the expression of LMP-1 was suppressed in OS tissues and overexpression of LMP-1 might serve as a tumor suppressor.

### 2.2. Restored LMP-1 Expression through Recombinant Adenovirus Infection

**Figure 2.**
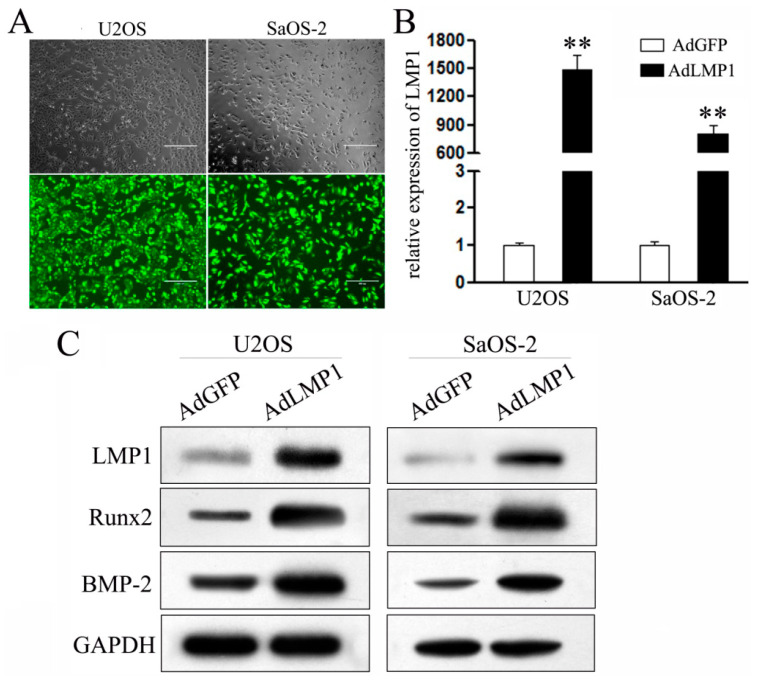
Restored *LMP-1* expression in OS cells through infection with recombinant adenovirus. (**A**) Fluorescence microscopy detected the green fluorescence protein upon infection. Eighty percent of OS cells showed strong fluorescence upon infection with the AdLMP-1 at 48 h upon infection (×100); (**B**) Real-time-PCR was performed to determine the mRNA level of *LMP-1* in OS cells infected with AdLMP-1 at MOI 10. Upon infection, the expression level of *LMP-1* mRNA was significantly upregulated compared with cells infected with AdGFP; (**C**) Western Blot assays were performed to explore the expression of LMP-1 protein after infection with AdLMP-1. The expression of LMP-1 combined with its downstream genes, Runx2 and BMP-2, were all upregulated upon infection. ** *p* < 0.01.

Subsequently, through infection with recombinant adenovirus AdLMP-1, we restored the expression of *LMP-1* in OS cell lines, U2OS and SaOS-2. Since the adenovirus has been recombined with the green fluorescence protein, cells infected with AdLMP-1 showed strong fluorescence (Figure 2A). Moreover, infection with AdLMP-1 significantly upregulated the mRNA level of *LMP-1* compared with cells infected with AdGFP adenovirus (Figure 2B). Consistently, further western blot assays identified that the protein levels of *LMP-1* combined with its downstream genes, bone morphogenetic protein-2 (*BMP-2*) and *Runx2*, were all upregulated in cells infected with AdLMP-1 (Figure 2C). These data indicated that AdLMP-1 induced overexpression of *LMP-1* and its downstream genes in OS cells.

### 2.3. Increased LMP-1 Inhibits Cell Proliferation and Cell Cycle Progression of OS Cells

**Figure 3.**
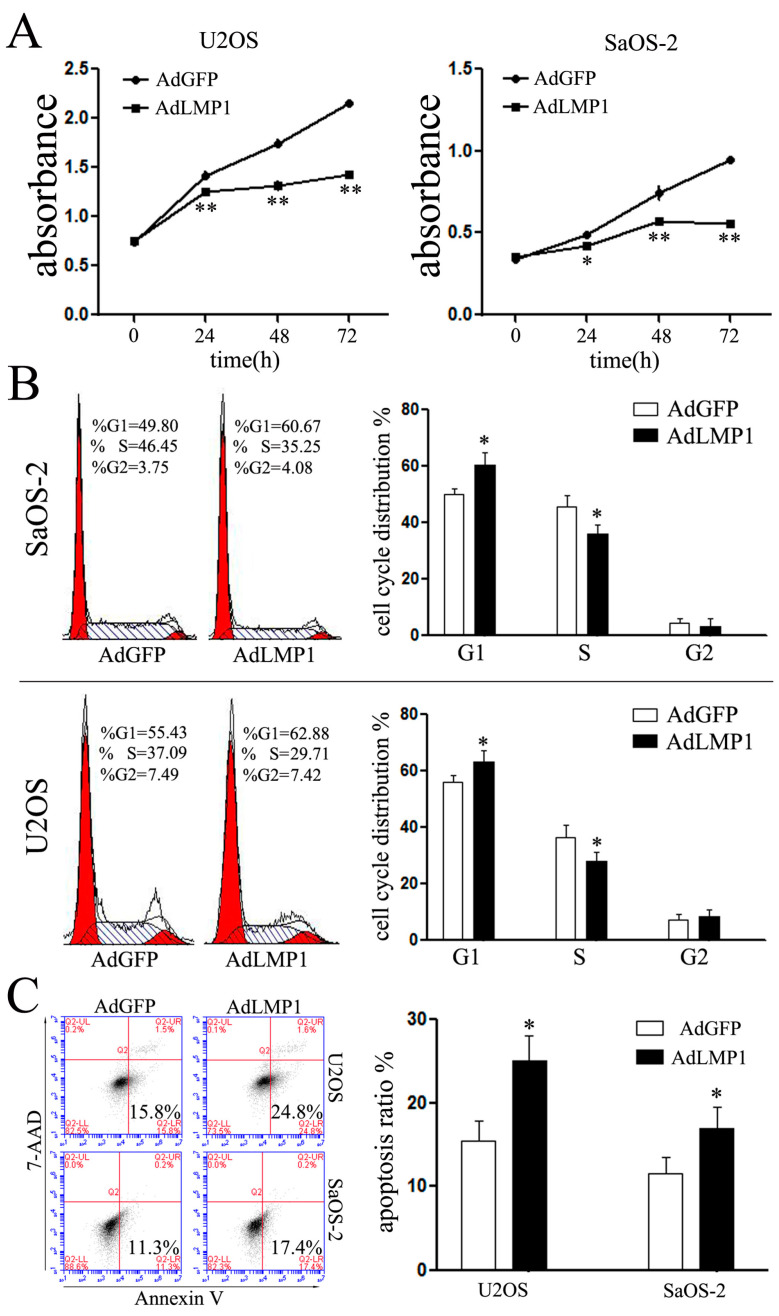
Overexpression of *LMP-1* suppressed OS cell growth. (**A**) CCK-8 assays were performed to determine the effects of *LMP-1* on OS cell proliferation. Overexpression of LMP-1 suppressed the cell proliferation of both OS cells; (**B**) Cell cycle analysis (**left**) and bar graphs (**right**) of U2OS and SaOS-2 cells by flow cytometry 48 h after transfection with LMP-1 adenovirus. Overexpression of *LMP-1* arrested cell cycle progression in G1 phase; (**C**) Apoptosis measurements (**left**) and bar graphs (**right**) in U2OS and SaOS-2 cells treated with LMP-1 and GFP adenovirus. Ectopic expression of *LMP-1* induced apoptosis of both cell lines. ** *p* < 0.01. * *p* < 0.05.

To explore the relevance of *LMP-1* and OS cell growth, cell proliferation rate was measured by CCK-8 assays. U2OS and SaOS-2 cells were treated with AdLMP-1 at MOI 10. Ectopic expression of *LMP-1* led to significant decrease in cell proliferation of both OS cells (Figure 3A). As proliferation directly linked to cell cycle distribution, the effects of *LMP-1* on OS cell cycle progression were analyzed. Overexpression of *LMP-1* resulted in an increased G1 population and a corresponding decrease in the S-phase population (Figure 3B). Furthermore, treatment with AdLMP-1 increased the percentage of early apoptotic cells as judged by PE Annexin V staining (Figure 3C). Taken together, these results indicated that *LMP-1* can efficiently inhibit cell proliferation and the cell cycle, and induce cell apoptosis *in vitro*.

### 2.4. Ectopic Expression of LMP-1 Inhibits Cell Migration and Invasion of OS Cells

**Figure 4.**
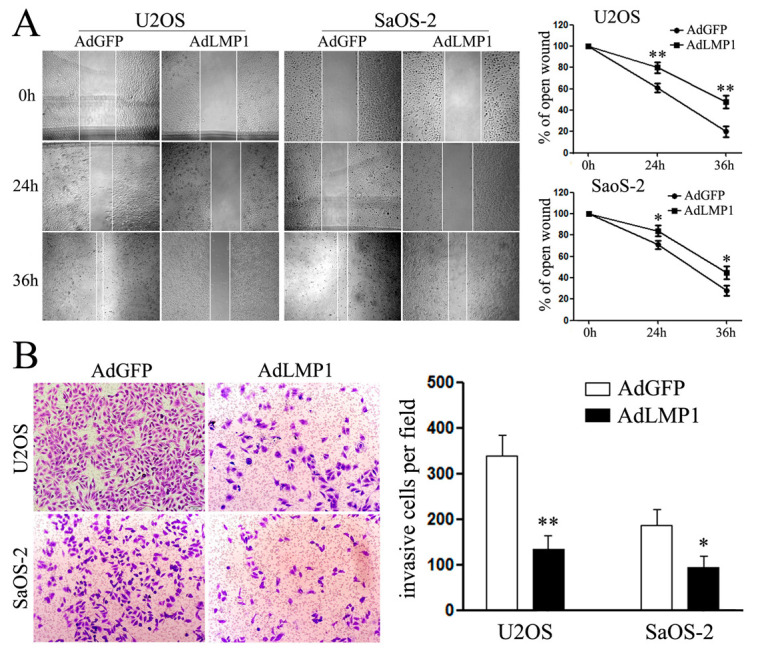
Overexpression of *LMP-1* suppressed OS cell migration and invasion. (**A**) Wound healing assays in U2OS and SaOS-2 cells treated with LMP-1 adenovirus for 48 h of infection. Overexpression of *LMP-1* resulted in a significant reduction in cell migration during the closing of the artificial wound; (**B**) Representative images (**left**) and bar graphs (**right**) depicting the invasion ability of U2OS and SaOS-2 cells after LMP-1 infection (×100). Ectopic expression of LMP-1 suppressed cell invasion of OS cells. ** *p* < 0.01; * *p* < 0.05.

Given that disease with pulmonary metastasis is the most common cause of death in OS patients [14], we investigated the effects of *LMP-1* on the migratory and invasive capacity of OS cells. We found that re-expression of *LMP-1* in OS cells resulted in a significant reduction in cell migration during the closing of an artificial wound created over a confluent monolayer (Figure 4A). Furthermore, the invasive capacity of OS cells transfected with AdLMP-1 was evaluated by Matrigel invasion chamber assays. AdLMP-1 dramatically inhibited the normally strong invasive capacity of both U2OS and SaOS-2 cells (Figure 4B). The results indicated that *LMP-1* could efficiently impair cell migratory and invasive capacity of OS cells *in vitro*.

## 3. Discussion

Although LMP-1 is a novel intracellular LIM domain protein shown to induce bone formation *in vitro* and *in vivo* [15], there are a limited number of published studies that have analyzed the expression and significance of *LMP-1* in the OS carcinogenesis. Herein, we found that the expression of *LMP-1* was suppressed in OS tissues and overexpression of *LMP-1* inhibited malignant phenotypes of OS cells. These results support the hypothesis that *LMP-1* functions as a tumor suppressor in OS.

Since firstly discovered by Boden in 1998, the role of *LMP-1* in osteoblast differentiation and bone formation has been widely investigated [8]. Located on chromosome 5q35.3, which includes 13 exons and 12 introns, the *LMP-1* gene contains a highly conserved PDZ domain in the *N*-terminal and three LIM domains in the *C*-terminal [8]. They identified that the expression of *LMP-1* could be found in bones of neural crest and mesoderm origin, as well as in both intramembranous and endochondral bone, which suggests that LMP-1 may be involved in a final common pathway of bone formation [8]. Since OS may be a kind of differentiation disease [5], we explored the role of *LMP-1* in OS. In this study, we found that the extent of LMP-1 immunoreactivity in OS tissues is limited. Of 43 total OS tissues, only 12 tumors (38%) were LMP-1 positive staining, while the majority showed no or weak staining. In contrary to the constitutive expression of *LMP-1* in bone formation, LMP-1 staining is largely absent in OS.

Previous work predominantly investigated the role of *LMP-1* in bone formation. Xu *et al.* found that combined (vascular endothelial growth factor) *VEGF* and *LMP-1* delivery enhances osteoprogenitor cell differentiation and ectopic bone formation [12]. Moreover, *LMP-1* overexpression in intervertebral disc cells leads to increased proteoglycan production *in vitro* and *in vivo* through a BMP-mediated mechanism [16]. However, the exact function of *LMP-1* in OS is less known. Thus, we performed a series of *in vitro* experiments. We restored the expression of *LMP-1* in two OS cell lines, U2OS and SaOS-2. Ectopic expression of *LMP-1* inhibited cell proliferation and invasion, arrested cell cycle progression and induced cell apoptosis of both OS cell lines, which further identified the role of *LMP-1* as a tumor suppressor in OS.

Then, what is the mechanism involved in LMP-1-mediated tumor suppression? Previous work reported that overexpression of *LMP-1* induced the mRNA expression of *Runx2* and *BMP-2* [16,17]. *Runx2* is considered to be one of the master regulators during the MSC osteoblast differentiation [18]. *Runx2* knockout is fatal in mice, which leads to a cartilaginous skeleton without any ossification and delayed chondrocyte maturation [19]. Otherwise, *BMP-2* belongs to the TGF-β superfamily of growth factors, which plays an important role in the induction of bone formation [20]. *BMP-2* is particularly important for the generation of mature osteoblasts *in vivo*, and induces the signaling cascades leading to cytoskeletal rearrangement [21]. Moreover, *BMP-2* has also been shown to be effective in stimulating proteoglycan production [22]. Considering the important role of osteoblast differentiation in OS, we measured the expression of both genes upon infection. The results showed that AdLMP-1 increased the expression of both *Runx2* and *BMP-2* in OS cells, which suggested that the osteogenetic roles of *Runx2* and *BMP-2* may be involved in LMP-1-mediated tumor suppression. However, further research is warranted to elucidate the mechanisms involved in *LMP-1*-mediated tumor suppression.

## 4. Material and Methods

### 4.1. Patients and Clinical Samples

Forty-three osteosarcoma specimens were collected before neoadjuvant chemotherapy in the Department of Orthopaedic Surgery and the Department of Oncology, the First Affiliated Hospital of Nanchang University from 2005 to 2010. The tumor samples have been confirmed by musculoskeletal pathologists. The matched normal tissues obtained from an area 5 cm distant from the tumor margin, were used as negative control. The study protocol and operational procedures were approved by the Human Ethics Committee of Nanchang University, and a signed informed consent form was obtained from all patients or patients’ family members for tissue biopsies.

### 4.2. Immunohistochemical Analysis

Four-micron-thick paraffin-embedded tissue sections were deparaffinized by xylene. Endogenous peroxidase activities were blocked by immersing these sections in methanol with 0.3% hydrogen peroxide for 30 min, followed by washing with water and phosphate (PBS). Then, antigen retrieval was made through boiling sections in citrate buffer. After non-specific binding was blocked with serum-free reagent for 20 min at room temperature, sections were incubated with anti-LMP-1 polyclonal antibody (Protein Tech Group, Chicago, IL, USA) at 4 °C overnight using predetermined optimal dilution (1:100). The slides were washed with water and PBS, and then incubated with the biotinylated secondary antibody for 30 min at room temperature. After washing with water and PBS, the peroxidase activity was revealed with 3,3′-diaminobenzidine (DAB+). The sections were counterstained with haematoxylin and photographed. Finally, the staining pattern was assessed quantitatively and also semi-quantitatively using the following scoring system: 0, no reactivity; + (weak), 1% to 10% positive cells; ++ (moderate), 10% to 50% positive cells; +++ (high), more than 50% positive cells.

### 4.3. Cell Line and Cell Culture

The human OS cell lines, U2OS and SaOS-2, were used in this study, which were cryopreserved in the laboratory of Orthopaedics Surgery at the First Affiliated hospital of Nanchang University (Nanchang, China). Cells were maintained in F-12 medium supplemented with 10% fetal bovine serum (FBS), 1% penicillin/streptomycin at 37 °C with 5% CO_2_.

### 4.4. Recombinant Adenovirus Vector Construction and Infection

The cDNA of human *LMP-1* was PCR amplified from K562 cells and subcloned into the adenovirus shuttle vector pAdtrack-CMV [23]. To increase the efficiency of infection, the Kozak sequence, GCCACCATGG, was introduced into the forward primer [24]. Then a *N*-terminal-(His)_6_-minimal sequence tag was added to the LMP-1 construct to facilitate identification of its expression by western blot assays. The recombinant adenovirus generation was carried out using the pAdEasy system as previously described [23,25]. The cells infected with AdGFP adenovirus were used as negative control. Adenovirus were produced in HEK-293A cells and amplified to obtain high titers, and the same procedures were applied for AdGFP. Through infecting U2OS and SaOS-2 cells, we detected LMP-1 expression by western blotting and also monitored infection efficiency by observed the level of green fluorescence protein. Before infection, 1 × 10^5^ cells were seeded into 6-well cell culture plates. After 6 h of culture, cells were infected with recombinant adenovirus vectors, AdGFP and AdLMP-1, at a multiplicity (MOI) of infection of 10 pfu (plaque-forming units)/cell as previously reported [23].

### 4.5. RNA Isolation and Quantitative Real-Time-PCR

U2OS and SaOS-2 cells were cultured for 5 days to reach confluence and their total RNAs were extracted using TRI Reagent according to the manufacturer’s instructions (Ambion Inc., Austin, TX, USA). Extracted RNAs were treated with DNase I (Invitrogen, Carlsbad, CA, USA) to remove chromosomal DNA. The quantity and purity of RNA products were respectively determined by optical density measurement at 260 nm and the ratio of measurements 260/280 and 260/230 nm (Nanodrop Technologies, Wilmington, DE, USA). Quantitative RT-PCR was performed using the Quanti-Tect SYBR Green PCR mixture (Applied Biosystems, Carlsbad, CA, USA) on an ABI PRISM 7900 Sequence Detection System (Applied Biosystems, Foster City, CA, USA). Specific primers for PCR reaction are: LMP-1-F: 5′-TAGCCAGTGTGGGAAGGTCCTGGAAGAG-3′; LMP-1-R: 5′-CTTCTTGCACTTGGCACAGCTGGGTG-3′, and the primers for GAPDH are: GAPDH-F: 5′-TCAACGACCACTTTGTCAAGCTCA-3′; GAPDH-R: 5′-GCTGGTGGTCCAGGGGTCTTACT-3′. The expression of GAPDH was used as internal control. Data were normalized to the internal control, and relative expression levels were elevated using the 2^−ΔΔ^*^C^*^t^ method. All experiments were repeated five times.

### 4.6. Proliferation Assay

Cell proliferation rate was detected by cell counting kit-8 (CCK8) assays according to the manufacturer’s instruction (Dojindo, Tokyo, Japan). Upon infection with AdGFP or AdLMP-1 48 h, U2OS and SaOS-2 cells were plated into 96-well culture plates (4000 cells per well). Then, 10 μL CCK8 buffer were added into each plates. After incubating for 2 h at the cell culture bin (thermo), the cell proliferation rate was detected at 0, 24, 48, 72 and 96 h after incubation, respectively. The absorbance of solubilized formazan crystals was read at 450 nm by a spectrophotometer microplate reader (Bio-Rad Laboratory, Hercules, CA, USA). The experiments were performed five times and showed as M ± SD for all different time points.

### 4.7. Cell Cycle Analysis and Apoptosis Assay

Cells cycle analysis was performed on U2OS and SaOS-2 cells 48 h after infection. Cells were harvested, washed twice with cold PBS, fixed in ice-cold 70% ethanol, incubated with propidium iodide (PI) and RNase A, and then analyzed by fluorescence-activated cell sorting (FACS). For analysis of cell apoptosis, U2OS and SaOS-2cells were collected and diluted to a concentration of 5 × 10^5^ cells/mL and washed two times with ice-cold PBS 48 h after infection. Cells were incubated with PE Annexin-V and 7AAD (BD Pharmingen, Franklin Lakes, NJ, USA) according to the protocol, and then analyzed by FACS. Cells undergoing early apoptosis bind only to Annexin V, and cells binding both are either in the late stages of apoptosis or already dead. All experiments were repeated three times.

### 4.8. Wound Migration Assay

In sub-confluent cells infected with AdGFP and AdLMP-1, the injury line was scratched with a tip 200 μL wide on cells plated in culture 6-well plates at 90% confluency. After being rinsed with phosphate-buffered saline, cells were allowed to migrate in medium without FBS, and photographs were taken (×40). These assays were taken in triplicate. Bright-field images of the field adjacent to the underline cross were taken at 0, 12, 24, and 36 h, respectively. Cell migration was then assessed by the cells across the gap.

### 4.9. Transwell Assays

The Transwell device containing microporous 8 μm membranes (Corning Incorporated, Corning, NY, USA) was placed in 24-well plates. The upper and lower chamber sides of the basal membrane were coated with 5 mg/mL of Matrigel (BD Biosciences, Bedford, MA, USA) and fibronectin, respectively. Two × 10^5^/mL cells were seeded in the upper chamber containing DMEM medium with 0.1% BSA. DMEM medium containing 10% FBS served as chemo-attractant and was added in the lower chamber. Invasion was followed for 24 h of infection. The cells adhering to the lower surface were fixed and stained with 0.1% crystal violet and transferred to a microscope slide. The total number of cells invading through the Matrigel (BD Biosciences, Bedford, MA, USA) was counted in six representative fields under microscopy (Olympus, Ariake, Tokyo, Japan).

### 4.10. Western Blot

Cells were harvested in ice-cold PBS 48 h after transfection and lysed on ice in cold modified radioimmunoprecipitation buffer supplemented with protease inhibitors. Protein concentration was determined by the BCA Protein Assay Kit (Bio-Rad, Segrate, Italy) and equal amounts of protein were analyzed by SDS-PAGE. Gels were electroblotted onto nitrocellulose membranes (Millipore, Billerica, MA, USA). Membranes were blocked for 2 h with 5% non-fat dry milk in Tris-buffered saline containing 0.1% Tween-20, and incubated at 4 °C overnight with primary antibody. Detection was performed by peroxidase-conjugated secondary antibodies using the enhanced chemiluminescence system (ECL) (Millipore, Billerica, MA, USA). Primary antibodies used were: anti-LMP-1, anti-Runx2 and anti-BMP-2 (cell signaling, Rockford, IL, USA) and GAPDH (Zhong-Shan JinQiao, Shanghai, China). The experiment was repeated three times.

### 4.11. Statistic Analysis

Data were delineated as M ± S.D., Differences between groups was analyzed using one-way ANOVA. The two-tailed Student’s *t*-test was applied to analyze the results for statistical significance when only two groups were compared. The statistical significance value was chosen as *p* < 0.05.

## 5. Conclusions

In conclusion, our findings demonstrate that *LMP-1* is suppressed in OS tissues and functions as a tumor suppressor in OS carcinogenesis. These findings not only advance our understanding of the molecular mechanism of OS carcinogenesis, but also suggest a strong rationale to further investigate *LMP-1* as a potential biomarker and therapeutic target for OS.
