# Correction: Liu et al. LIM Mineralization Protein-1 Inhibits the Malignant Phenotypes of Human Osteosarcoma Cells. *Int. J. Mol. Sci.* 2014, *15*, 7037–7048

**DOI:** 10.3390/ijms27083576

**Published:** 2026-04-17

**Authors:** Huiwen Liu, Lu Huang, Zhongzu Zhang, Zhanming Zhang, Zhiming Yu, Xiang Chen, Zhuo Chen, Yongping Zen, Dong Yang, Zhimin Han, Yong Shu, Min Dai, Kai Cao

**Affiliations:** 1 Department of Orthopedics, the First Affiliated Hospital of Nanchang University, Nanchang 330006, China; 2 Department of Orthopedics, the First People’s Hospital of Jingdezhen, Jingdezhen 333000, China; 3 Department of Children Health and Care, Jiangxi Maternal and Child Health Hospital, Nanchang 330006, China; 4 Department of Oncology, the First Affiliated Hospital of Nanchang University, Nanchang 330006, China

In the originally published article [1], an error was identified in Figure 4A. Upon reviewing the raw experimental data, the authors noted discrepancies in the SaOS-2/AdLMP1 0 h. In the SaOS-2/AdLMP1 0 h panel, a narrow strip of cells appears duplicated. It is speculated that this artifact occurred due to the inadvertent duplication of a layer during image processing (specifically while drawing lines on the image). Corrected Figure 4A is provided below.

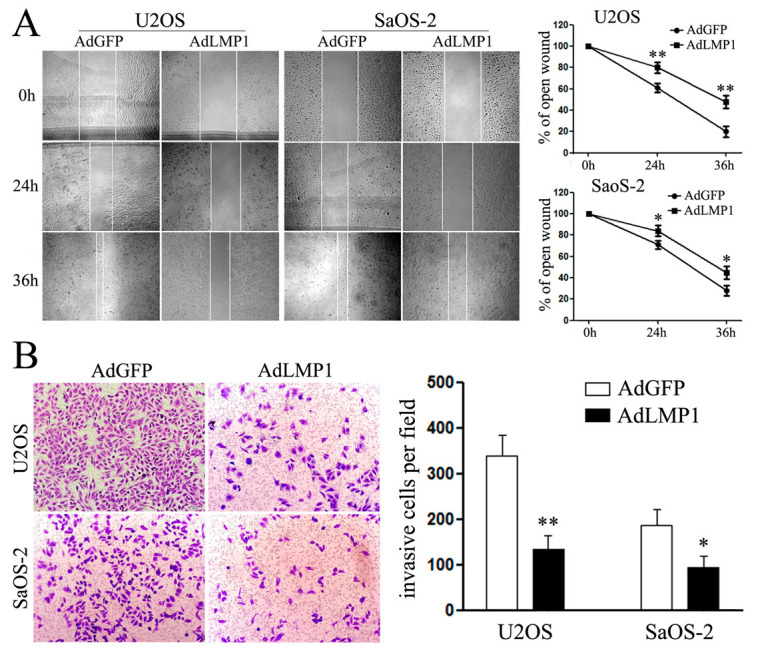



The authors state that the scientific conclusions are unaffected. This correction was approved by the Academic Editor. The original publication has also been updated.
